# Hematological and Biochemistry Profile and Risk Factors Associated with Pulmonary Tuberculosis Patients in Guyana

**DOI:** 10.1155/2016/6983747

**Published:** 2016-04-12

**Authors:** Rajini Kurup, Keon Flemming, Sudish Daniram, Shenika Marks-James, Roberta Roberts Martin

**Affiliations:** Faculty of Health Sciences, University of Guyana, Turkeyen Campus, Georgetown, Guyana

## Abstract

*Objective.* To evaluate the hematological and biochemistry profile of patients with or without HIV-TB at the Georgetown Chest Clinic, Guyana.* Methods.* An observational, laboratory based study was designed to assess the relationship of PTB and HIV with patients routine biochemical and hematological values. The study was conducted during the period January 2013 to December 2014; a total sample size of 316 patients was enrolled following exclusion and inclusion criteria.* Results.* Mean age of study population was 40.1 ± 13.8 (95% CI 38.6–41.7) and most were between 40 and 49 age group (27.8%, 95% CI 23.2–33.0). More males were in the study 74.4% (95% CI 69.3–78.8) than females 81% (95% CI 21.1–30.7). 30% (95% CI 25.3–35.3) had a sputum smear grade of 3+ and 62.5% (95% CI 47.0–75.7) showed a CD_4_ count <200. The study demonstrated significantly low hemoglobin (Hb) 91.7% (95% CI 78.2–97.1), low WBC 27.8% (95% CI 15.8–44.0), high indirect bilirubin 7.4% (95% CI 2.1–23.3), ALT 41.8% (95% CI 28.4–56.7), and AST 72.2% (95% CI 57.3–83.3) among TB-HIV patients. Homelessness RR (relative risk) 2.2 (95% CI 0.48–12.3), smoking RR 1.09 (95% CI 1.01–1.19), and gender (male) RR 1.2 (95% CI 0.61–2.26) were main associated risk factors.* Conclusions.* There is slight variation among PTB and PTB-HIV coinfected patients in some hematological and biochemistry parameters.

## 1. Introduction

Clinical TB remains a major global health problem and the second leading cause of death from an infectious disease worldwide. TB has a global incidence of 8.6 million, a mortality of 1.3 million, and sadly a global treatment success rate of only 87% [1]. Studies are carried out on TB and HIV in almost every aspect so as to develop new or improved systems in the early diagnosis and control of the comorbidity. The laboratory markers associated with PTB with or without HIV coinfection play a major role, although a nonspecific one, in the diagnosis and prognosis of TB in patients [2, 3]. The demonstrable changes that PTB + HIV, PTB, and HIV seropositive patients have in their hematological and metabolic values all in all showed varied pictures in each study as a result of the phase of the infection and the causative agents, namely,* Mycobacterium tuberculosis* and* Human Immune Deficiency Virus* [2].

Guyana, a lower-middle-income country being ranked as one of the TB high-burden countries in the Americas, sustains a tuberculosis (TB) prevalence (including HIV) of 131 for every 100,000 persons in 2012 [1]. In Guyana, 28% of tested TB patients are diagnosed as HIV coinfected [4]. The country is very vulnerable to the devastating impact of HIV/AIDS due to the most productive age groups (15–49 years) in society being affected with 1.4% in 2013 [5].

Tuberculosis and HIV are devastating infectious diseases associated with a low socioeconomic status, with the reputation of bringing stigma and discrimination to their victims and inflicting economic losses to low- and middle-income nations [6, 7]. Both drug resistance and HIV worsen the TB disease burden among individuals and are a threat to global TB control. Although nondefinitive, some routine clinical laboratory assays can be useful in assisting diagnosis and prognosis at a low cost, but care must be taken while interpreting them due to other confounding factors such as drug induced or viral hepatitis, diabetes coinfection, and age.

The purpose of this study is to observe the hematological and biochemical status of pulmonary tuberculosis (PTB) patients with or without Human Immune Deficiency Virus (HIV) coinfection at the Chest Clinic, Georgetown Public Hospital, Guyana.

## 2. Methods

### 2.1. Study Site and Population

An observational, laboratory based retrospective descriptive study was designed to assess the relationship of PTB and HIV with patients routine biochemical and hematological values. The study was conducted during the period January 2013 to December 2014 at Georgetown Chest Clinic, Guyana. Sample sizes of 316 patients were enrolled following exclusion and inclusion criteria.

*Inclusion Criterion.* All patients who were diagnosed of PTB and PTB + HIV with no past history of TB were considered for the present study.

*Exclusion Criteria.* Patients with the following conditions were excluded from the study:

1. patients under inspection;
2. patients with any type of hepatitis complications at the time of PTB diagnosis;
3. inadequate medical records to allow complete analysis;
4. pregnancy at time of PTB diagnosis;
5. patients with a clinical history of chronic renal failure;
6. hemoglobinopathy;
7. neoplastic disease and collagen vascular disease;
8. deceased patients upon follow-up;
9. malaria and worm infested PTB diagnosis;
10. sputum sample taken after initiation of treatment.

### 2.2. Laboratory Testing

Patients suspected of pulmonary tuberculosis were tested using fluorescence microscopy and treatment begins as soon as PTB is confirmed. An HIV test is done, if patients HIV status is unknown at the time and a RBS is taken to screen for diabetes. CD4 count and sputum smear grade were also investigated. Hematology and biochemistry tests of the patients were performed at the Georgetown Public Hospital Cooperation (GPHC) Laboratory. Parameters evaluated for biochemistry were serum BUN, serum creatinine, serum electrolytes (K^+^, Cl^−^, and Na^+^), aspartate aminotransferase (AST), alanine aminotransferase (ALT), direct bilirubin, total bilirubin, and indirect bilirubin. Hematology parameters evaluated were fasting blood sugar (FBS), hemoglobin concentration, total white blood cell (WBC), and platelet count. Voluntary counselling and general physical evaluation of all the patients were carried out at the center. The patients were then asked questions to determine risk factors, habits, and sociodemographic data (age, gender, ethnicity, education, smoking history, and alcohol intake).

### 2.3. Ethical Considerations

The study was approved by the University of Guyana, Guyana. Permission was also obtained from the Institutional Review Board (IRB), Ministry of Health, Guyana, to proceed with the study. Individual patients were assigned numbers for identification.

### 2.4. Statistical Analysis

All pieces of information were first entered in Excel spreadsheet and later transferred to SPSS 20.0 and JMP for further analysis.

## 3. Results

### 3.1. Patient Selection Profile

A total of 495 TB patients that were not under investigation were selected by the National Tuberculosis Program (NTP) staff. Of these, 37 had Extrapulmonary Tuberculosis (EPTB), 23 had both pulmonary tuberculosis (PTB) and EPTB, 1 had liver disease, 3 females were pregnant, 10 had malaria within a year, before TB diagnosis, and 105 had too little or no lab test available.

### 3.2. Background Characteristics

A total of 316 patients with newly diagnosed pulmonary TB were selected for the study, 235 (74.4%, 95% CI 69.3–78.8) were males and 81 (25.6%, 95% CI 21.1–30.7) were females (*p* < 0.001). The mean age ± SD of subjects was 40.2 ± 13.8 years (95% CI 38.6–41.7). Median age and standard error (SE) and interquartile range (IQR) of the study population were 40 ± 0.77 and 20, respectively (Table 1).

Of the total 316 patients, 44 (13.9% 95% CI 10.5–18.2) were HIV positive and 272 (86.1%, 95% CI 81.8–89.5) were HIV negative with *p* > 0.05. Among those coinfected with PTB-HIV, a significant proportion (62.5%, 95% CI 47.0–75.7) had a CD4 count <200 (*p* < 0.001). A significant higher number (30.0%, 95% CI 25.3–35.3) of patients had a sputum grade of 3+ (*p* < 0.001) (Table 2).

### 3.3. Hematology Results

Tables 3 and 4 show the number (percentage), mean, and 95% CI of the hematological parameters among the study population. Significant high parameters were found among TB-HIV coinfected population.

### 3.4. Biochemistry Results

Table 5 shows the number (percentage), mean, and 95% CI of biochemical parameters among the study population. Most biochemistry parameters had significant high values among TB-HIV coinfected population.

### 3.5. Risk Factors

Table 6 demonstrates the different risk factors associated with the study population. Patients at particular risk of the TB/TB-HIV were males and homeless. Significant risk factors demonstrated within the study population were diabetes 14.9% (95% CI 11.4–19.3), HTN 3.5% (95% CI 1.9–6.1), allergy 2.8% (95% CI 1.5–5.3), homelessness 0.09 (95% CI 0.03–2.7), being alcoholic 34.2% (95% CI 29.2–39.6), and smoking 32.9 (95% CI 27.9–38.7) (*p* < 0.001).

## 4. Discussion

This study was a first-time effort to relate hematological and biochemistry parameters among TB patients with or without HIV at the Georgetown Chest Clinic, Guyana. Though this study did not have enough TB-HIV coinfected patients, a concrete conclusion cannot be made.

Our study reveals that men were most infected than females and 51% of patients being between the ages of 30 years and 49 years which is a normal trend seen in most parts of the world [8, 9]. This study demonstrated significant high number with CD_4_ > 200. CD_4_ and CD_8_ T-cells are important immune components against TB and HIV and reduction in the count could suppress the immunity of the patient [10].

Some studies agree that the mean of the total WBC count in PTB patients is usually normal or not significantly elevated as compared to the normal mean of a population [11, 12]. However, Amilo et al. state that there is a statistically higher total WBC count among PTB and PTB-HIV comorbid patients, where neutrophilia is the main contributor to the increase [2]. Platelets are effector cells that play an important role in the inflammatory and immunological response and have the capacity to release cytokines and chemokines, thus acting as an immune regulator [13]; therefore this direct relationship between platelet and WBC is logical because when there is an immune response at PTB infection, platelets tend to increase.

This study did not show any variation on blood sugar although Bailey and Grant [6] found that the relationship that exists between diabetes and tuberculosis has even been shown to be more significant than that of HIV/AIDS with TB, although it is not consistently reproduced in different populations [14].

Risk factors play a major role in the magnitude of TB and HIV. There were many risk factors involved in this study, but the magnitude of TB was high among the homeless, smokers, and males. Socioeconomic status is always a major risk factor [15].

There are several limitations that might have affected our conclusions; first, analysis was done based on the data available from the Chest Clinic. Second, a large-scale cross-sectional study might have produced a better conclusion on the laboratory parameters. Finally, other risk factors could have also included in this study.

## 5. Conclusion

Hematological and biochemistry parameters are important, simple, and cheaper method in analyzing the pattern of health status among PTB patients with or without HIV.

## Figures and Tables

**Table 1:** Characteristics of study population in the Georgetown Chest Clinic, Guyana.

Characteristics	Total population *n* = 316	95% CI	TB 272 (86.1)	95% CI	TB-HIV 44 (13.9)	95% CI
Age (mean ± SD)	40 ± 13.8	38.6–41.7	40.0 ± 14.3	38.3–41.7	40.9 ± 10.7	37.7–44.2
Age group						
<20	20 (6.3)	4.1–9.6	20 (7.4)	4.8–11.1	0	
20–29	59 (18.7)	14.8–23.3	52 (19.1)	14.9–24.2	7 (15.9)	7.9–29.3
30–39	74 (23.4)	19.1–28.4	57 (21.0)	16.5–26.2	17 (38.6)	25.7–53.3
40–49	88 (27.8)	23.2–33.0	77 (28.3)	23.3–33.9	11 (25.0)	14.6–39.4
50–59	45 (14.2)	10.8–18.5	39 (14.3)	10.7–19.0	6 (13.6)	6.4–26.7
>60	30 (9.5)	6.7–13.2	27 (9.9)	6.9–14.1	3 (6.8)	2.3–18.2
		**p** < 0.0001		**p** < 0.0001		**p** < 0.05
Gender						
Male	235 (74.4)	69.3–78.8	201 (73.9)	68.4–78.7	34 (77.3)	63.0–87.1
Female	81 (25.6)	21.1–30.7	71 (26.1)	21.2–31.6	10 (22.7)	12.8–36.9
		**p** < 0.0001		**p** < 0.0001		**p** < 0.001
Ethnicity						
Afro-Guyanese	131 (41.5)	36.2–47.0	101 (37.1)	31.6–43.0	30 (68.2)	53.4–80.0
Amerindian	33 (10.4)	7.5–14.3	31 (11.4)	8.1–15.7	2 (4.5)	1.3–15.1
Indo-Guyanese	71 (24.5)	18.2–27.4	68 (25.0)	20.2–30.5	3 (6.8)	2.3–18.2%
Mixed	77 (24.4)	20.0–29.4	68 (25.0)	20.2–30.5	9 (2.0)	2.0–11.1%
Portuguese	4 (1.3)	0.5–3.2	4 (1.5)	0.5–3.7	0.0	
		**p** < 0.0001		**p** < 0.0001		**p** < 0.001

**Table 2:** Hematological parameters among TB and TB-HIV patients.

	HIV serostatus				
	Negative% (*n*)	95% CI	Positive% (*n*)	95% CI	Total% (*n*)
Hb					
Low	80.2% (200)	74.9–84.8	91.7% (33)	78.2–97.1	81.8% (233)
Normal	19.6% (49)	15.2–25.0	0.08% (3)	2.8–2.2	18.3% (52)
	**p** < 0.0001		**p** < 0.0001		
WBC					
High	17.0% (42)	12.8–22.2	2.8% (1)	0.5–14.2	15.2% (43)
Low	5.3% (13)	3.1–8.7	27.8% (10)	15.8–44.0	8.1% (23)
Normal	77.7% (192)	72.2–82.5	69.4% (25)	53.1–82.0	76.7% (217)
	**p** < 0.0001		**p** < 0.0001		
PLT					
High	27.3% (30)	19.8–36.2	12.5% (2)	3.5–36.0	25.6% (32)
Low	4.5% (5)	1.9–10.2	6.3% (1)	1.1–28.3	4.8% (6)
Normal	68.2% (75)	58.9–76.1	81.3% (13)	56.9–93.4	69.6% (87)
	**p** < 0.0001		**p** < 0.0001		
Blood sugar					
High	37.7% (91)	31.8–44.0	3.1% (12)	1.9–4.7	36.9% (103)
Low	4.5% (11)	2.5–7.9	0.3% (1)	0.04–13.4	4.3% (12)
Normal	57.7% (139)	51.4–63.7	65.7% (25)	49.8–78.7	58.8% (164)

**Table 3:** Mean and SD of hematological parameters among TB and TB-HIV patients.

	Reference value	HIV positive		HIV negative	
		Mean ± SD	95% CI	Mean ± SD	95% CI
Blood sugar					
FBS (mg/dL)	70–105				
Male		95.3 ± 16.9	(68.3–122.2)	121.3 ± 79.9	(96.6–146.0)
Female		0		118.9 ± 58	(87.9–149.8)
*p* value		<0.001		<0.001	
RBS (mg/dL)	70–120				
Male		122.6 ± 47.4	(103.1–142.2)	132.1 ± 75.1	(119.7–144.6)
Female		101.4 ± 14.6	(90.2–112.6)	123.7 ± 66.3	(104.6–142.7)
*p* value		<0.001		<0.001	(7.7–8.7)
WBC	4–11 (10^3^/uL)	5.5 ± 1.7	(4.6–6.3)	8.2 ± 3.5	
*p* value		<0.001		<0.001	
PLT	150–450 (10^3^/uL)	260.4 ± 146	(194.4–326.4)	376.3 ± 173.3	(343.6–409.1)
*p* value		<0.001		<0.001	

**Table 4:** Biochemistry parameters among TB and TB-HIV patients.

	Reference value	HIV positive		HIV negative	
		Mean ± SD	(95% CI)	Mean ± SD	(95% CI)
Indirect bilirubin (mg/dL)	0.1–1.0	0.4 ± 0.3	0.4–0.6	0.41 ± 0.25	0.36–0.44
T-bilirubin (mg/dL)	0.2–1.2	0.7 ± 0.3	0.59–0.79	0.68 ± 0.7	0.58–0.77
D-bilirubin (mg/dL)	0.0–0.5	0.26 ± 0.2	0.20–0.31	0.24 ± 0.24	0.21–0.27
AST (IU/L)	5–34	64.6 ± 61.9	45.5–83.6	37.9 ± 38.2	33.2–42.6
ALT (IU/L)	4–36	45.6 ± 49.9	30.2–60.9	35.2 ± 45.8	29.6–40.8
Cl (mmol/L)	98–107	101.5 ± 4.7	99.4–103.6	101.1 ± 4.4	100.4–101.7
Na (mmol/L)	135–148	136 ± 3.4	134.8–137.8	137.7 ± 3.4	137.1–138.2
K (mmol/L)	3.5–5.3	4.2 ± 0.6	3.9–4.4	4.2 ± 0.4	4.1–4.3
Creatinine (mg/dL)	0.4–1.4	0.96 ± 0.43	0.83–1.1	1.0 ± 1.5	0.8–1.2
BUN (mg/dL)	7–18	12.4 ± 5.6	10.6–14.1	11.7 ± 5.3	11.0–12.3

**Table 5:** Mean and SD of biochemistry parameters among TB and TB-HIV patients.

	HIV serostatus				
	Negative% (*n*)	95% CI	Positive% (*n*)	95% CI	Total% (*n*)
Indirect bilirubin					
High	2.8% (4)	1.1–7.2	7.4% (2)	2.1–23.3	3.6% (6)
Normal	97.1% (134)	92.7–98.8	92.5% (25)	76.6–97.9	96.4% (159)
T-bilirubin					
High	5.4% (12)	3.1–9.2	5.0% (2)	1.4–1.7	5.4% (14)
Normal	94.6% (209)	90.7–96.7	95.0% (38)	83.5–98.6	94.6% (247)
D-bilirubin					
High	1.7% (4)	0.6–4.3	7.1% (3)	2.5–19.0	2.5% (7)
Normal	98.3% (230)	95.7–99.3	92.8% (39)	80.9–97.5	97.5% (269)
AST					
High	35.2% (91)	29.9–41.2	72.1% (31)	57.3–83.3	40.5% (122)
Normal	64.7% (167)	58.7–70.3	27.9% (12)	16.7–42.7	59.5% (179)
ALT					
High	23.3% (60)	18.5–28.7	41.8% (18)	28.4–56.7	25.9% (78)
Normal	76.7% (198)	71.2–81.5	58.1% (25)	43.3–71.6	74.1% (223)
Cl					
High	1.9% (3)	0.06–5.6	0.6% (1)	0.08–21.7	2.3% (4)
Low	17.86% (27)	12.6–24.7	2.9% (5)	10.1–43.4	18.5% (32)
Normal	80.1% (121)	73.0–85.7	9.3% (16)	51.8–86.8	79.2% (137)
K					
High	1.3% (2)	0.03–4.7	4.5% (1)	0.08–21.7	1.7% (3)
Low	1.7% (27)	1.2–2.5	22.7% (5)	10.1–43.4	4.1% (7)
Normal	80.7% (122)	73.7–86.3	72.7% (16)	51.8–86.8	94.2% (163)
Na					
High	1.3% (2)	0.03–4.7	4.5% (1)	0.08–21.7	
Low	17.9% (27)	12.6–24.8	22.7% (5)	10.1–43.4	20.2% (35)
Normal	80.8% (122)	73.7–86.3	72.7% (16)	51.8–86.8	79.8% (138)
Creatinine					
High	8.6% (22)	5.7–12.7	11.6% (5)	5.1–24.5	9.10%
Low	0.04% (1)	0.006–0.2	0.0% (0)	0	0.3% (1)
Normal	90.9% (232)	86.8–93.9	88.3% (38)	75.5–94.9	90.6% (270)
BUN					
High	8.4% (21)	5.6–12.5	9.5% (4)	3.7–22.0	8.6% (25)
Low	8.8% (22)	8.8–13.0	9.5% (4)	3.7–22.0	8.9% (26)
Normal	82.7% (206)	77.5–86.9	80.9% (34)	66.6–90.0	82.5% (240)

**Table 6:** Risk factors associated with TB patients with HIV positive and negative.

		HIV positive	HIV negative	OR (95% CI)	RR (95% CI)
Diabetes	Yes	3 (0.95%)	44 (14.0%)	0.38 (0.11–1.27)	0.42 (0.13–1.29)
	No	41 (13.0%)	227 (72.1%)		

HTN	Yes	0	11 (3.5%)		
	No	44 (13.9%)	261 (82.6%)	0	

Allergy	Yes	0	9 (2.9%)		
	No	44 (13.9%)	263 (83.2%)	0	

Homelessness	Yes	1 (0.3%)	2 (0.6%)		
	No	43 (13.6%)	270 (85.4%)	3.14 (0.28–35.4)	2.4 (0.48–12.3)

Being alcoholic	Yes	13 (4.1%)	95 (30.1%)		
	No	31 (9.8%)	177 (56.0%)	0.78 (0.4–1.6)	0.81 (0.44–1.48)

Smoking	Yes	9 (2.9%)	95 (30.1%)		
	No	35 (11.1%)	177 (56.0%)	0.48 (0.22–1.04)	1.09 (1.01–1.19)

Substance abuse	Yes	6 (1.9%)	42 (13.3%)		
	No	38 (12.0%)	230 (72.8%)	0.86 (0.34–0.22)	0.89 (0.39–1.97)

TB contact	Yes	9 (2.9%)	55 (17.4%)		
	No	35 (11.1%)	217 (68.7%)	1.0 (0.46–2.24)	1.0 (0.5–2.0)

Gender	Female	10 (3.2%)	71 (22.5%)		
	Male	34 (10.8%)	201 (63.6%)	1.20 (0.56–2.56)	1.2 (0.61–2.26)
